# Psychosocial and socioeconomically aspects of mothers having a child with cleft lip and/or palate (CL/P): a pilot-study during the first year of life

**DOI:** 10.4317/jced.56288

**Published:** 2020-09-01

**Authors:** Konstanze Scheller, Jasmin Urich, Christian Scheller, Stephan Watzke

**Affiliations:** 1 Department of Oral and Maxillofacial and Facial Plastic Surgery, Martin-Luther-University Halle-Wittenberg (Head: apl. Prof. Dr. Dr. A.W. Eckert), Ernst-Grube-Straße 40, 06120 Halle, Germany; 2 Department of Neurosurgery, Martin-Luther-University Halle-Wittenberg (Head: Prof. Dr. C. Strauss), Ernst-Grube-Straße 40, 06120 Halle, Germany; 3 Department of Psychiatry, Martin-Luther-University Halle-Wittenberg (Head: Prof. Dr. D. Rujescu), Julius-Kühn-Str.7, 06112 Halle/Saale

## Abstract

**Background:**

The emotional impact on parents at the birth of their new-born with cleft lip and/or palate (CL/P) can be traumatic for parents, especially mothers, and affect the sensitive early parent-child relationship. Unlike many other congenital malformations facial deformities are visible to all. The uncommon facial appearance creates feelings and reactions in the mother, families and other people. Only few studies deal with this psychosocial burden of these mothers.

**Material and Methods:**

This pilot-study deals with mothers’ early experiences (n=84) having a child with CL/P. Mothers were asked to complete a questionnaire at diagnosis, birth and after lip surgery. The questions were focused on the social background of the mother (educational degree, marital status, lifestyle and prenatal care), the medical information at diagnosis and the following reaction. The surveys were administrated from 01/2014 – 12/2016.

**Results:**

84 mothers of affected children (CL/P) replied the completed questionnaire (84/103, 81.5%). At diagnosis 65 mothers (77%) lived in a solid partnership and 44% worked full-time (40h). The diagnosis caused fear among the mothers (60.7%, p≤0.01), despair (27.4%, p≤0.01), grief (17.9%, p≤0.01) and guilt (16.7%, p≤0.01). Despite the emotional stress after the diagnosis only 5 mothers asked for psychological support (6.0%). The medical information by the gynecologist (41.6%) or maxillofacial surgeon (32.2%) was rated as “good” (n=26) or “very good” (n=26) in 60.2%. A lack of medical information and care was rated with “insufficient” (11.9%) or “poor” (14.3%).

**Conclusions:**

There are only few studies about mothers’ early feelings and emotions having a child with a CL/P. We found high parental stress, physical and emotional strain among the mothers after diagnosis, mostly caused due to insufficient information’s. This stress was not correlated with the educational level and CLP appearance showed no relation about the socioeconomic status.

** Key words:**Cleft lip/palate, mother’s emotional experience, psychosocial aspects

## Introduction

Orofacial clefts are one of the most common birth defects in humans (1,2). In Europe EUROCAT data show an upper confidence interval of the basic prevalence (2005 to 2016) of 8.85 / 100.000 neonates (http://www.eurocat-network.eu) with a clear north-south gradient.

The etiology of orofacial clefts is still unclear and is considered to be multifactorial caused by an interaction of environmental and genetic factors (3). About 70% of cases with CL/P are isolated occurring without any additional structural or cognitive abnormalities (3). These birth defects create a high burden of disease owing on their complexity. Accompanied with a large number of problems for the child like speech disorder, hearing deficit, chronic ear infections, dental and palatal deformities are also psychosocial problems of both, the mothers (parents) and the child (4). Coy *et al.* (5) found that some mothers of children with CL/P showed extraordinary protectiveness and responsiveness towards their children, as they viewed them as vulnerable. However, this behavior again leads to a psychological burden on the mother, the child and the social environment. Results of another study indicate that mothers and fathers of children with oral clefts may differ in their psychosocial adjustment and that mothers may overall experience more psychosocial problems than fathers (6). The psychological impact may even negatively affect the early mother-child relationship.

In contrast to many other congenital malformations, these orofacial malformations are visible at first look to the baby. Parents, and therefore especially mothers’ of a child with cleft lip and palate (CL/P) are confronted with psychosocial and emotional problems caused by the behavior of the social environment (7). Across countries and cultures, parents’ feelings of shock, anger, grief and worry have been documented in surveys and in qualitative studies (8,9). These experiences may extend from the time when parents first know about their child’s diagnosis (whether during pregnancy or at delivery) through childhood.

The goal of treatment for CL/P is to improve appearance, speech and psychosocial function (10) and so usually, therapy for CL/P is multidisciplinary involving the surgical, orthodontic, phoniatrics and pediatric disciplines. It is suggested that appropriate medical care in Cleft Centre may lead to improvement in quality of life for families with CL/P children as seen in many studies before (11).

## Material and Methods

-Aim

The aim of this pilot-study was to evaluate the psychological and social aspects in mothers having a cleft child at three different times during the first year of life. The first year of life was chosen because surgical reconstruction of the cleft lip was there performed. Especially emotional and social experiences of the mothers should be analyzed.

-Study sample

This pilot-study was performed from 01/2014 – 12/2016 and included mothers of children with a visible non-syndromic orofacial cleft, like cleft lip (CL) or cleft lip and palate (CLP). Mothers of children with a syndromic cleft combined with other malformations were excluded from this study. All mothers and their child were regularly cared for in the same hospital and all surgical procedures were done at this University hospital.

The Human Research Ethics Committee of the Martin-Luther-University Halle-Wittenberg provided ethical approval to conduct this research (ethics committee processing number: 2014-12). All standard ethical procedures, which included study information sheets, voluntary participation, informed consent, confidentiality and anonymity in the management of data and reporting of study findings, were adhered.

-Concept of Reconstructive Surgery

In all patients, primary reconstruction of the upper lip and the hard and soft palates was completed during the first 2 years of life. At an age of 4-6 months the lip-closure according to Pfeifer was done (12,13). Therefore mothers and children stayed for 3-4 days in hospital. At 7th postoperative day the extraoral sutures, even absorbable sutures, in the upper lip were removed. Veloplasty according to Furlow (14) and hard palate reconstruction were done simultaneous at the age of 15-18 month.

-Data Acquisition and Instruments

The applied anonymous questionnaires, the study information sheet and the informed consent were in German language and send to the mothers of an affected child by mail. The attending mother was instructed to answer the questionnaire and to return by mail.

Inclusion criteria were a child with non-syndromic cleft lip and or palate (CL/P) and knowledge of written and spoken German. Exclusion from the study was done if the child presented a syndromic cleft, like a CHRAGE-syndrome or any other chromosomal aberration, like trisomy 21.

-Measures

Validated and standardized measures were used in this pilot-study. Therefore an anonymous self-reported questionnaire was used. These measures included: social background, educational degree and employment of the mother, prenatal preventive care, access of medical information, evaluating the attractiveness and the reactions of the social environment to the child before and after lip reconstruction.

-Statistical analysis

The information gained from the anonymous personal data acquisition was collected in a database and analyzed statistically (SPSS 12.0; SPSS Corp, Chicago, IL). The results were analyzed using the Wilcoxon signed-rank test and the Wilcoxon rank-sum test (Mann-Whitney-U-test) to compare analyses of 2 paired patient samples. A parametric distribution of the independent data was not expected. The Kruskal-Wallis tests and the chi-quadrat test was used for comparative analyses of more than 2 patient samples on a significance level of *p* = 0.05.

## Results

-Patients and Clinical Data

In total, 84 of 103 asked mothers participated in this study and answered the questionnaires completely, representing 81.6%. Nonresponses were not related to a special type of cleft, sex, age or any irregularities during cleft treatment. 45 cleft children were female (53.6%) and 39 male (46.4%). In total, 60 children experienced cleft lip and palate (CL/P, 71.4%) and 24 patients experienced isolated cleft lip (CL, 28.6%).

-Social background

**Figure 1:**
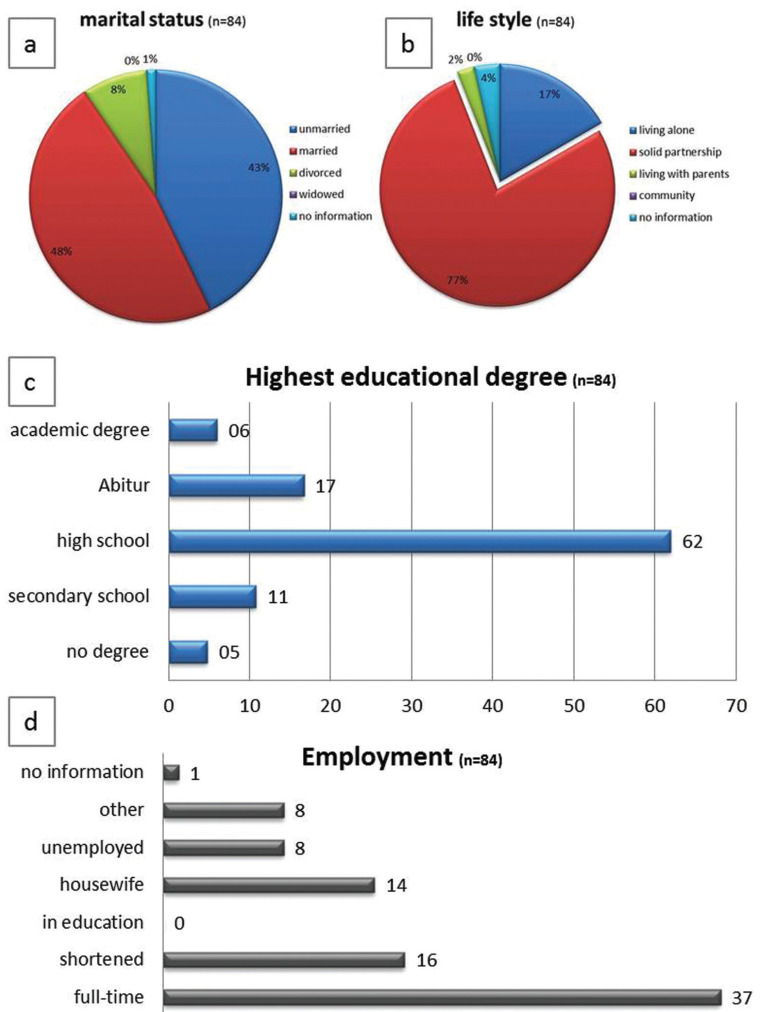
The diagram shows the martial status (a), the life style status (b), the highest educational degree (c) and the employment situation (d) in interviewed mothers of a cleft child (n=84).

48% of the mothers (n=40) were married and 43% mothers (n=36) not (unmarried, Fig. 1a). In total 77% of the mothers (n=65) lived in a solid partnership and only 14 mothers (17%) lived alone as a single mother with the cleft child (p≤0.01, Fig. 1b). Only one mother (1.2%) did not answer this questionnaire about the martial status and 2 mothers (2.4%) did not give any information about the actually life style (listed as “no comment”).

-Educational degree 

The highest school leaving certificate of mothers included in this study follows the similar distribution in the society (Fig. 1c). Most mothers (n=52, 61.9%) graduated from high school, 9 from secondary school (10.7%), 14 graduated with Abitur (16.7%) and 5 graduated from university (6.0%). The educational degree among the mothers corresponds to the population structure in the state examined (16).

-Employment of the mother

Very astonishing in our evaluation is that most of the mothers of the cleft children (n=37, 44.0%) worked full-time with 40 hours a week at time of diagnosis (Fig. 1d). 19.0% of these mothers worked shortened and the employment rate was around 2/3 (63.0%). This is certainly due to the regional circumstances (formerly Eastern Germany) and the associated work-culture, and surveys in other parts of the former West Germany will still bring different results today.

-Prenatal preventive care 

82 of 84 mothers (97.6%) took preventive care and regular provided check-up during pregnancy. 27 mothers (32.1%) said that the diagnosis of the cleft manifestation of their child was given during the regularly prenatal examination (ultrasound examination by the supervising gynecologist). 22 from 27 mothers (81.4%) reported about a “good” (n=8, 29.8%) and 14 about a “very good” medical information at time of prenatal diagnosis the diagnosis (51.9%).

The use of no pre-examination or only rare preventive examinations was denied. Nevertheless 54 mothers (64.3%), respectively parents, were informed about the diagnosis of an orofacial cleft at time of birth. 20.2% of the mothers, who were informed at birth, was given information about the diagnosis by the gynecologist, 23.8% by an maxillofacial surgeon, 10.7% by the midwife and 9.5% of the mothers was given no information.

Medical information at time of diagnosis

**Figure 2:**
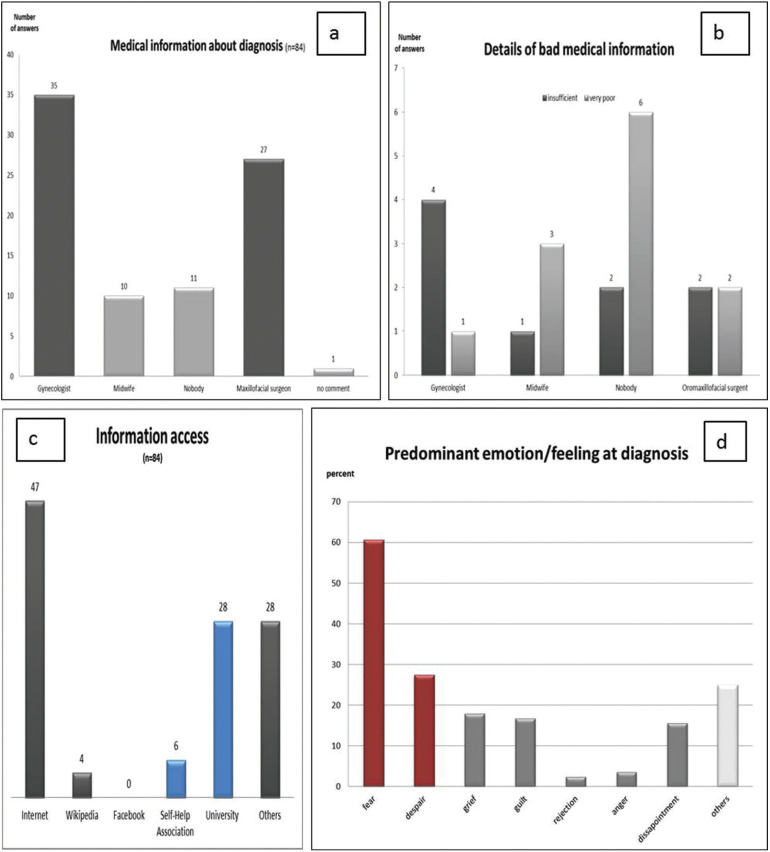
First medical information. Most mothers were informed about the consequences of the diagnosis of a cleft lip and palate (CL/P) by a gynecologist or a maxillofacial surgeon (a). The “bad” information (b), the access of medical information after diagnosis (c) and the predominant feelings (red bar) and emotions (grey bar) at diagnosis were analyzed in detail (d).

At the time of diagnosis the parents, especially the mothers (n=84), were informed about the diagnosis and the treatment options of a CL/P by different medical disciplines (Fig. 2a). Most mothers were informed by the attending gynecologist or obstetrician (n=35, 41.6%) and on second by an maxillofacial surgeon (n=27, 32.2%). 30.1% each rated the quality of medical information as “very good” (n=26,) and “good” (n=26). So about 2/3rd (60.2%) of the mothers felt well informed.

9 mothers mothers felt not very comfotable with the primary information at the time of diagnosis and described it as “satisfactory” (10.7%).

In total, 21 mothers (25%) described the individualised counselling as “insufficient” (n=10, 11.9%) or “non-existent” (n=12, 14.3%) at the time of diagnosis. 5 mothers who complained about an “insufficient” (11.9%) medical information at time of diagnosis received the diagnosis at birth (n=5, 6%) or at the pre-natal examination (n=4, 4.8%). One mother gave no information in this regard. On demand, who was responsible for the “poor” information 5 mothers stated the attending gynecologist and 2 mothers the consulting oromaxillofacial surgent.

12 mothers (14.3%) did not even receive any medical information about the postnatal care and therapy options. All these mothers (n=12) were given the diagnosis of the CL/P at birth. There was no medical information by the attending medical staff (Fig. 2b). Six mothers even reported that there was no one who felt responsible for any medical information (50%) at birth.

-Additional information

56.0% of all mothers, that were confronted with this diagnosis, obtained additional information about cleft lip and palate visiting the internet (n=47). The information gathering through the internet was used by all mothers and in this study especially from mothers with high school graduation (30 from 52, 57.7%). However, they did not give any details about the websites visited.

Then the special and personally consultation in a maxillofacial department of the universities nearby followed (n=28, 33.3%). But significantly (*p*=0.003) more information about CL/P was gathered using the internet than attending a specialized university. Social media like Facebook (n=0, 0.0%) and others, and self-help organizations (n=6, 7.1%) were not attended for information acquisition. In summery besides the general, unfiltered and impersonal information from the internet especially specialized universities were personally visited for further information (Fig. 2c).

-Predominant feelings and emotions at diagnosis

Predominant feelings/impressions and emotions concerning the mother when they received the diagnosis of a child with CL/P were fear (60.7%) and despair (27.4%). The most listed emotions were grief (17.9%) and guilt (16.7%), while emotions like rejection (2.4%) and anger (3.5%) were rare (Fig. 2d). Despite the severe bad feelings and the emotional distress that the mothers reported after the diagnosis, only 5 mothers asked for psychological support (6.0%). To what extent this was due to a lack of offer, was not specified in detail.

In this context the time of diagnosis is very important. 66.7% of the mothers who were informed about the diagnosis at birth (n=54, 100%) reported about the predominant feeling fear. Even if the quality of medical information was “very good” and “good” the dominant feeling of fear dominated. This fear did not depend on the quality of medical information (28 mothers rated the information “very good” and “good”).

## Discussion

The relationship between socioeconomic status of the mother and the risk of a congenital malformation of the child has always been controversial (16,17). There are some studies that describe greater risk of non-chromosomal anomalies with increasing socioeconomic deprivation, except orofacial clefts (18). Other reports have been fairly consistent in reporting a higher prevalence in lower social classes for oral clefts, particularly for cleft palate (16). In our study the school leaving certificate of mothers followed the general distribution of educational degrees to the population structure in the state examined (15). There was no socioeconomic class that was more correlated with the appearing of CL/P. While some articles in literature show that the mother’s education level represents a significant risk factor associated with CL/P occurrence, this relationship could not be seen in our study population.

77% of the asked mothers (n=65) lived in a solid partnership, while only 48% were married. These social background data were compared with the general population in the state (19). According to state statistic there were more partners married (59%), than seen in the interviewed mothers. With a percentage of 43 unmarried mothers of children with CL/P this rate is twice as seen in the national comparison (24.3%). All in all in our study population the marriage and solid partnership is almost equated.

Communicating the diagnosis of a visible malformation like a CL/P in the unborn child during a prenatal examination, or at birth of the child, represents an emotionally stressful situation for all involved. In this study only 1/3rd of the participants (32.1%) were informed about the manifestation of a CL/P during prenatal examination (ultrasound by the attending gynecologist), even if 97.6% of the mothers went to all regular pregnancy examinations. This prenatal detection rate is by ultrasound examination is a bit higher than the values according to the observations of Robbins *et al.* (20) who described the prenatal diagnosis efficacy of orofacial clefts with 20%. Further the prenatal ultrasound technique has developed fast during the last years and allows almost 100% detection of CL/P at the end of the 1st trimester of pregnancy. The manifestation of the lip (CL) alone can usually only been seen during the 2nd trimester in ultrasound examination (21). While 81.4% of mothers were very satisfied with the information they received at the time of pre-natal diagnosis, there were even 4 mothers complaining about “insufficient” information at this time. This “insufficient” information was given in all cases by the investigating gynecologist. Even if it is only a small part of patients (14.8%) who received this “insufficient” information about the unborn child, this can be decisive for the later mother-child relationship. The prenatal diagnosis of an orofacial cleft is a challenge for both, the mother and the physicians; it allows the mothers to become prepared for the special needs of an infant with a cleft (20).

Parents and especially mothers of children with congenital malformations often describe feelings like fear, guilt, self-blame and associated anxiety (8,11). Exactly these feelings have also been seen in our study group. To understand the main difference of feelings and emotions, the main definition has to be respected. Feelings are basic physical senses, while emotions are products of a complex state of feelings caused by mental processes. The main emotion at time of diagnosis was grief as a multifaceted reaction to the loss of something what might include others like anger, guilt, anxiety, sadness, and despair.

64.3% of the interviewed mothers reported, that the diagnosis of the CL/P was given to them at birth. This seems to be an inappropriate time point and might explain the predominant feeling of fear (60.7%) that was mainly described by the mothers. Actually the methods in ultrasound imaging have improved so far that mothers (parents) will be prepared about the diagnosis before birth. So the prenatal counseling will give parents the change to take help from specified surgeons and be prepared (17). Few guidelines exist for practitioners who wish to meet patient and family expectations for clear and caring communication (20,22).

In summery our study showed that there was no socioeconomic status or level of mothers’ educational level that was associated with the birth of a child with CL/P. The worse time for medical information was the time of birth. No matter who did the medical information, a dedicated and informative conversation could help the mothers and parents the most. The prenatal diagnosis may be shocking for the mother, but respectively the time until birth can be used for further information to handle the negative feelings and emotions and preparations and organization.
